# Management of Delayed Complications of Hydrogel Scleral Buckles

**DOI:** 10.3390/jpm12040629

**Published:** 2022-04-14

**Authors:** Hsin-Yu Yang, Wei-Kuang Yu, Chieh-Chih Tsai

**Affiliations:** 1 Taipei Veterans General Hospital Yuanshan and Suao Branch, Yilan 264018, Taiwan; lisa771102@yahoo.com.tw; 2 Department of Ophthalmology, Taipei Veterans General Hospital, Taipei 11217, Taiwan; hikaruyu@gmail.com; 3 School of Medicine, National Yang Ming Chiao Tung University, Taipei 11221, Taiwan

**Keywords:** hydrogel scleral buckles, retinal detachment, glaucoma, anterior orbitotomy

## Abstract

(1) Background: hydrogel scleral buckles (HSB)-related complications can happen decades after implantation, although this material has been retrieved for a long time. Due to its fragile texture, ensuring the complete removal of this material and avoiding complications are challenging. Incomplete removal, iatrogenic complication, recurrent retinal detachment, and infection could occur. (2) Methods: chart review of patients who developed delayed HSB-related complications and received removal of HSB in Taipei Veterans General Hospital from 2004 to 2021. The presenting symptoms, prior diagnosis before referral, clinical findings, image features, surgical technique, operative findings, and outcome were analyzed. Detailed surgical procedure and tips for removal were demonstrated in the study. (3) Results: a total of eleven patients were identified. The presenting symptoms include limitations to extraocular movement (ten eyes, 90.9%), ocular redness (eight eyes, 72.7%), ocular fullness (eight eyes, 72.7%), pain (six eyes, 54.5%), and exposed ocular foreign body (five eyes, 45.5%). Of note, six patients (54.5%) have monocular glaucoma and four of them have intractable high intraocular pressure. All patients underwent surgeries to smoothly remove swollen HSB via transcutaneous or transconjunctival approach. Most symptoms improved after surgery and no cases developed surgical-related complications. (4) Conclusions: although HSB have been off the market for decades, delayed complications are still emerging. Clinicians should remain alert for potential complications for patients with prior HSB surgeries. Early diagnosis and meticulous management can help to safely remove the expanded HSB and reduce the associated complications.

## 1. Introduction

Hydrogel scleral buckles (HSB) were first introduced as an alternative to silicone buckles for treatment of rhegmatogenous retinal detachment in the 1980s and are thought to possess better softness and elasticity. Softness increase comfort and decreases the rate of scleral erosion and protrusion. Elasticity and mild hydration of hydrogel in the tissue could fill the dead space between the eyeball and the surrounding tissue. In addition, this material in the past was thought to absorb and slowly release antibiotics, further preventing bacterial growth [1]. Complications associated with this material have been reported due to hydrophilic degradation, causing swelling, extrusion, intraocular intrusion, strabismus, infection, bony erosion, and even globe loss [2,3,4,5,6,7,8,9]. It was gradually removed from the market in the 1990s.

Many patients developed HSB-related complications decades after implantation. Some of the cases were initially diagnosed with thyroid-associated orbitopathy, idiopathic orbital fibrosis, orbital tumors, conjunctival cysts, or neurological disorders [4,6,10,11]. Removal of this brittle material was challenging due to its fragile characteristics and the surrounding adhesion to ocular tissue due to fibrosis. The perforation rate during surgery can be as high as 18% [3]. For surgeons who were unfamiliar with this material, incomplete removal, recurrent retinal detachment (up to 29.4%), scleral rupture (up to 17.6%) and other surgical associated complications can occur [3,6].

In this study, we investigated the various clinical manifestations of delayed complications of swollen HSB and shared our surgical techniques to avoid the severe complications associated with manipulations.

## 2. Materials and Methods

We collected all patients with late HSB complications who underwent surgery for HSB removal in Taipei Veterans General Hospital, a tertiary medical center, from 2004 to 2021. Age, sex, time interval between HSB implantation and removal, presenting symptoms, initial diagnosis, clinical findings, surgical technique, intraoperative findings, and outcome were retrospectively reviewed.

Intraocular pressure (IOP) change was defined as the change in IOP before surgery and one month after surgery. The reason we chose IOP one month postoperatively for comparison is that post-operative tissue edema usually subsides at this time. IOP reduction was defined as a >20% IOP decrease compared to pre-operative IOP.

Location of HSB was categorized into groups according to the relative location to the globe equator in orbit-computed tomography (CT): anterior-located and posterior-located HSB. Anterior-located HSB were defined as more than 50% of HSB coverage anterior to the globe equator in sagittal cut, while the others were defined as posterior-located HSB.

SPSS statistical software version 22.0 (IBM corp., Armonk, NY, USA) was used for data management and statistical analysis. Categorical and paired continuous data were compared using the Fisher’s exact test and Wilcoxon Signed ranks test, respectively.

## 3. Results

**Figure 1:**
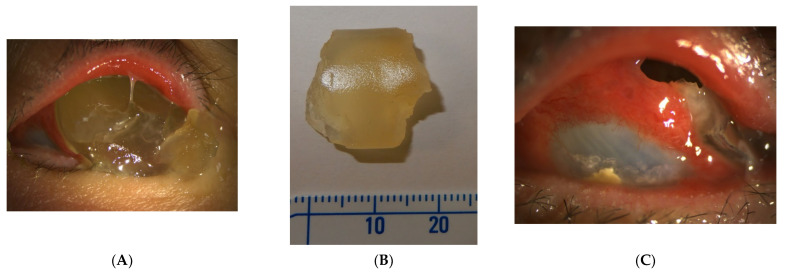
(**A**) A case of bulbar atrophy after retinal detachment repair with hydrogel scleral buckles. The picture of the left eye showed protrusion of a yellowish, jelly-like material from the orbit, with compression of the eyeball to the medial orbit. (**B**) A yellowish and soft hydrogel scleral buckle dropped from the patient’s eye. (**C**) After the foreign body fell out, a hole was left in the superotemporal orbit. The atrophic eyeball was seen, and the surrounding residual hydrogel scleral buckles were noted.

**Figure 2:**
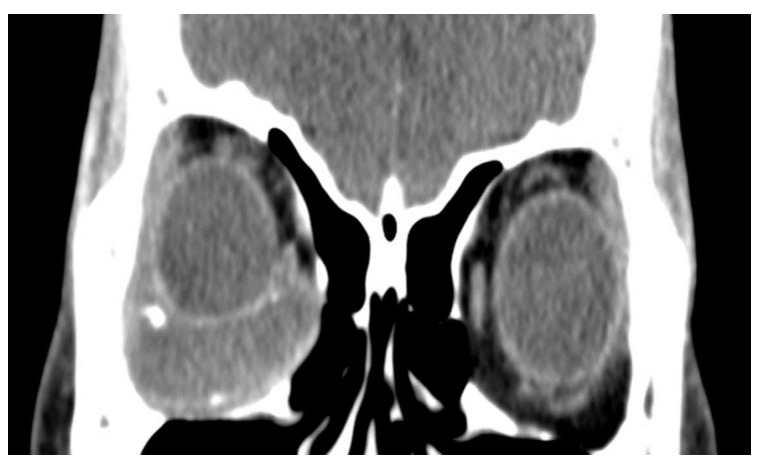
Orbital CT showed a homogenous and hypointense mass with scattered hyperintense spots. This finding was compatible with hydrated hydrogel scleral buckles and dystrophyic calcification.

**Figure 3:**
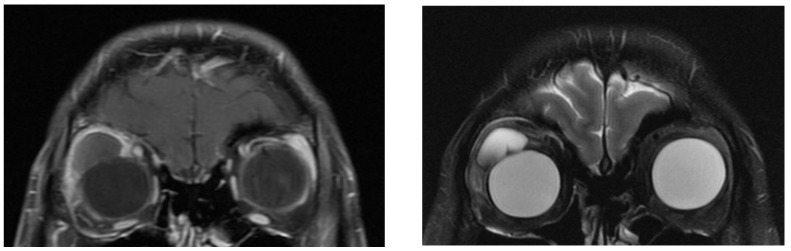
MRI of hydrogel scleral buckles were hypointense in T1 (**left**) and hyperintense in T2 (**right**) images.

**Figure 4:**
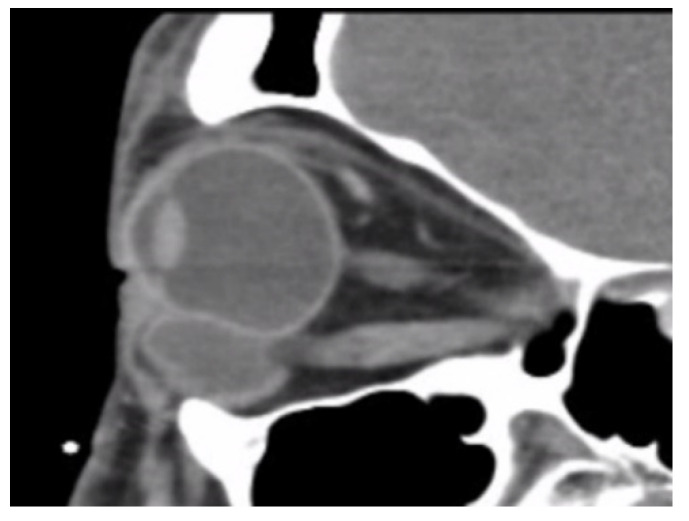
Orbital CT showed anteriorly located hydrogel scleral buckles.

**Figure 5:**
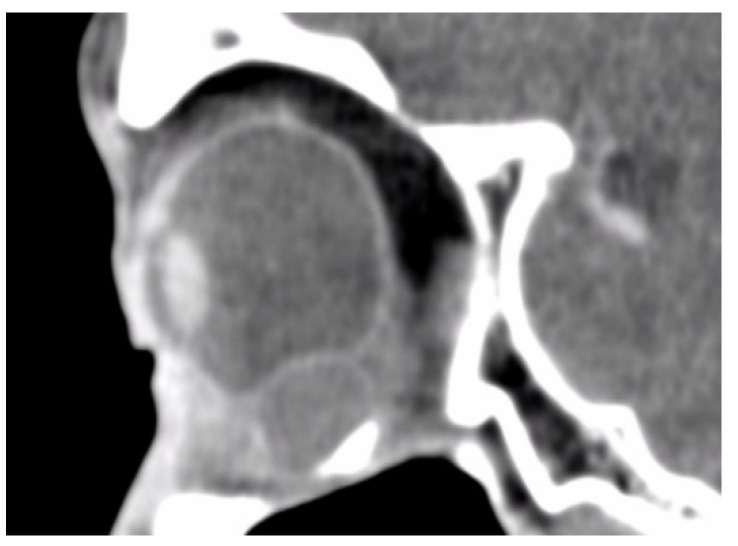
Orbital CT at sagittal view revealed posteriorly located hydrogel scleral buckles.

In total, we collected eleven cases; Table 1 summarizes the clinical data. One patient had bulbar atrophy before the diagnosis of swollen HSB (Figure 1). Table 2 shows the presenting symptoms of the patients. The symptoms included limitation of extraocular movement (ten eyes, 90.9%), ocular redness (eight eyes, 72.7%), ocular fullness (eight eyes, 72.7%), pain (six eyes, 54.5%), and exposed ocular foreign body (five eyes, 45.5%). Six patients (54.5%) received a correct diagnosis when referred to the medical center. Initial diagnoses other than swollen HSB included eyeball rupture (one eye, 9.1%), and orbital tumor (four eyes, 36.4%). Six patients (54.5%) had glaucoma in the diseased eyes and were treated with at least one combined form or two antiglaucoma medications before orbital HSB swelling was found. Four patients (66.7%) were male, but there was no significance of sex and glaucoma correlation according to Chi-Square analysis (*p* = 0.652). In the six patients with glaucoma, four cases had inadequate IOP control and one case received minimally invasive glaucoma surgery due to uncontrolled IOP one year before HSB removal. New onset of limitations of extraocular movement prompted the doctors to survey for orbital lesions. The typical CT image finding of HSB is a circumferential and homogenous mass around the globe. As this material usually swells when it absorbs water and forms a pseudocapsule around itself, CT images show an isointense signal with a vitreous and non-infiltrative lesion that deforms the eyeball. A scattered, and hyperintense signal, which corresponds to calcific change, can also be found around the swollen HSB (Figure 2). In MRI study, the HSB usually show hypointensity in T1-weighted images and hyperintensity in T2-weighted images, as these materials absorb water (Figure 3). According to the location of HSB in orbital CT, we categorized the HSB coverage into two groups: anterior-located (Figure 4) and posterior-located HSB (Figure 5). In the six patients with glaucoma, four (66.7%) showed posterior-located HSB. However, Fisher’s exact test of the relationship between the posterior-located HSB and glaucoma diagnosis did not reach clinical significance (*p* = 0.061).

**Figure 6:**
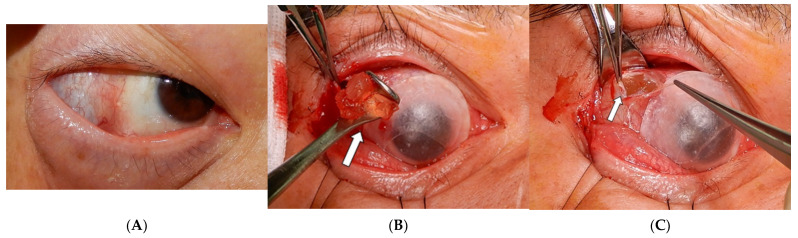
(**A**) The photo showed protrusion of hydrated hydrogel scleral buckles. (**B**) After lateral canthotomy, removal of the hydrogel scleral buckles with a Desmarres lid retractor (the white arrow) was performed through one conjunctival wound. (**C**) The pseudocapsule (the white arrow) is usually found enclosing the hydrogel scleral buckles.

All cases’ HSB were finally removed through one external conjunctival wound if the HSB could be easily approached anteriorly, or through one skin wound if the HSB were posteriorly located. Table 3 summarized the surgical approach of the study cases. Lateral canthotomy was suggested to better expose the surgical field. This jelly-like hydrogel was fragile and can easily be broken into pieces in an attempt to remove it. We used blunt instruments such as muscle hooks or a Desmarres lid retractor to scoop out rather than grab the swelling buckles (Figure 6A,B). A pseudocapsule was usually found during the dissection of orbital space (Figure 6C). Surgeons preferred not to dissect further, as removal of the HSB inside the pseudocapsule was adequate.

At the end of surgery, irrigation of the empty space with antibiotics and steroids was performed to prevent infection and decrease inflammation, respectively. The HSB were safely removed in all patients. Evisceration was performed in addition to HSB removal in one case due to severe orbital cellulitis. In the surgical field, no implants were found other than HSB. No intraoperative scleral rupture was noted. There was no endophthalmitis, recurrence of retinal detachment, delayed bleeding or associated complications found in the follow-up period after surgery.

After removal of the HSB, ocular symptoms improved in most cases (Table 2). However, a small residual degree of eye movement limitation was found in two cases. No patients received further strabismus surgery. In the six glaucoma patients, four of them showed IOP improvement one month after HSB removal. There was no change of antiglaucoma medication during this period. However, there was no statistical significance of IOP change before and after HSB removal in the ten cases using Wilcoxon signed ranks test (*p* = 0.189, Table 4).

## 4. Discussion

HSB absorb tissue fluids and progressively expand over the decades. The swelling of hydrogel itself has a mass effect on the orbital area, causing compression of the globe. HSB can cause inflammation and fibrosis of the surrounding tissue, and a pseudocapsule usually forms to enclose the hydrogel. If the material distends to a greater extent, it can intrude into the sclera or conjunctiva, or extrude from the orbit [2,4,10]. Delayed HSB complications associated with implant swelling and protrusion are usually found from 54 to 284 months after surgery [3]. The indications to remove the implant include pain, ocular discomfort, ocular inflammation, cosmetic concerns and diplopia [3,6]. In our patient group, the most common symptom was limitations of extraocular movement. The most severe complication of HSB in our study was the marked extension of hydrogel within the orbit, leading to compression, extrusion, and orbital cellulitis in one case. Ocular redness was thought to be caused by an immune reaction to the exposed foreign body. Strabismus was possibly due to myotoxicity, adhesion and scarring [12]. Strabismus can develop after all kinds of scleral buckle implants in around 5–25% of patients in the long term [12,13]. However, HSB-related strabismus has been reported in up to 29% of patients in the previous literature, probably due to its evident mass effect, displacing the ocular content and muscles [3]. As expanded HSB can cause a distinct bulky effect and more severe ocular motility disturbance, the removal of expanded HSB is helpful in reducing the extent of diplopia and the following strabismus surgery.

Incorrect initial diagnosis can lead to incomplete removal and increase iatrogenic complications for unprepared surgeons [4,10]. In our study, four patients (36.4%) were initially diagnosed as having an orbital tumor when they were referred to us. Surgeons who were not familiar with HSB removal may not be able to remove this fragile material safely. The rate of unexpected perforation during surgery ranges from 5.3% to 17.6% [3,6,7]. A comprehensive exam of the retina and supplemental retinal laser may be needed before the operation. Recurrent retinal detachment can occur in from 5.3% to 29.4% of patients after HSB removal [3,6,7]. Endophthalmitis, corneal edema, glaucoma, and scleral necrosis were also reported as complications of removal surgery [6,7].

A proper surgical approach is essential to extract the brittle hydrogel smoothly and avoid disturbing ocular structures. Although the previous literature mentions extensive peritomy, a cryo-assisted approach [14], suction traction [15], or the use of boric acid [16], we use a relatively simple means of removing HSB. An extensive peritomy can result in scleral perforation when dissecting the periocular tissue. Further adhesion and scarring of tissue could ensue. Compression of the globe during manipulation may cause recurrent retinal detachment while the HSB are removed. In contrast, we make a relatively small wound around the skin or fornix and lateral canthotomy to better expose the surgical field. This external approach near the orbital ring can further make a tunnel to the target implant. Usually, the implant is enclosed by a pseudocapsule. The jelly-like material can be extracted with blunt instruments such as a Desmarres lid retractor or muscle hook through the tunnel (Figure 6). At the end of surgery, irrigating the space with steroids and antibiotics may decrease the degree of inflammation and rate of infection.

It is worth mentioning that none of the previous studies mentioned glaucoma status or refractory IOP before removal of the implant. In our study, six patients had glaucoma, and four of them had inadequate IOP control. There are two possible explanations for this. First, these patients had a longer interval between HSB implantation surgery and removal, ranging from 26 to 30 years. Previous large-population studies documented the interval between implantation and removal from 8 to 23 years [3,6,17]. With an extended period in our study, pathological changes in periorbital tissue such as fibrotic change, impaired ocular circulation and ocular compression, can play a role in glaucomatous development. Another explanation is that the relative location and degrees of HSB coverage may influence the development of glaucoma. Four out of the six cases with glaucoma in our study showed HSB coverage posterior to the equator. Compared to the anterior location, posteriorly located HSB cannot release the swelling pressure through protrusion of the orbital exit. Previous studies documented the degrees of HSB coverage but not the relative location to the equator. Roldán-Pallarés et al. followed up 415 cases with hydrogel implantation for up to seven years [17]. More than half of the patients received HSB coverage over 180 degrees, and 45% of the patients had 360-degree encircling HSB coverage. None of these patients reported elevated IOP or glaucoma status, but unspecified orbital fullness was noted in only six cases. These may imply that the relative location compared to the globe equator, not the range of HSB coverage, may be the key factor that influences IOP. The relationship between IOP elevation and the location of swelling HSB must be proven through further studies.

## 5. Conclusions

Although HSB had not been used for more than 30 years, delayed complications associated with this material still emerged in recent years. Patients could present with various clinical manifestations. A detailed history, examination, and image findings are the keys to correct diagnosis and proper removal of the implant. In our study, an external approach was proved to be a safe way to remove HSB. Blunt instruments are suggested to prevent inadvertent perforation. Early diagnosis and meticulous management are essential to avoid severe sequelae in these patients.

## Figures and Tables

**Table 1:** Patient characteristics.

Characteristics	Total *n* = 11
Mean age, years (range)	51 (43–82)
Male, *n* (%)	7 (63.6)
Mean interval between SB implant and explant, years (range)	20.45 (14–30)
Correct initial diagnosis, *n* (%)	6 (54.5)
Glaucoma, *n* (%)	6 (54.5)
Location of SB relative to equator in CT image, *n* (%)	
Posterior-located	4 (36.4)
Anterior-located	7 (63.6)
Coverage of SB, *n* (%)	
180 degrees	8 (72.7)
120 degrees	2 (18.2)
90 degrees	1 (9.1)

CT: computed tomography, SB: scleral buckles.

**Table 2:** Symptoms before and after hydrogel scleral buckles removal.

Symptoms	Before SB Removal	After SB Removal
Pain, *n* (%)	6 (54.5)	1 (9.1)
Redness, *n* (%)	8 (72.7)	1 (9.1)
Swelling sensation, *n* (%)	8 (72.7)	1 (9.1)
EOM limitation, *n* (%)	10 (90.9)	2 (18.2)
Exposed foreign body, *n* (%)	5 (45.5)	0 (0.0)

EOM: extraocular movement, SB: scleral buckles.

**Table 3:** Surgical approach.

Surgical Approach	Total *n* = 11
Operation under general anesthesia, *n* (%)	10 (90.9)
Operation under retrobulbar anesthesia, *n* (%)	1 (9.1)
Transconjunctival approach, *n* (%)	9 (81.8)
Transcutaneous approach, *n* (%)	2 (18.2)
Evisceration, *n* (%)	1 (9.1)

**Table 4:** Intraocular pressure before and one month after HSB removal surgery.

Case Number (Medication)	IOP before Surgery (mmHg)	IOP after Surgery (mmHg)
1	Not applicable due to bulbar atrophy before surgery	
2 (G-alphagan, IZBA, carteolol)	27	27
3 (G-cosopt, xalatan)	19	15
4 (G-cosopt)	28	21
5 (G-lumigan, timolol, trusopt)	35	34
6 (G-duotrav)	8	6
7	15	15
8	14	15
9	13	14
10	12	14
11 (G-alphagan, cosopt, IZBA)	34	19

G: glaucoma case.

## Data Availability

Not applicable.
